# Day-to-day fasting glycaemic variability in DEVOTE: associations with severe hypoglycaemia and cardiovascular outcomes (DEVOTE 2)

**DOI:** 10.1007/s00125-017-4423-z

**Published:** 2017-09-15

**Authors:** Bernard Zinman, Steven P. Marso, Neil R. Poulter, Scott S. Emerson, Thomas R. Pieber, Richard E. Pratley, Martin Lange, Kirstine Brown-Frandsen, Alan Moses, Ann Marie Ocampo Francisco, Jesper Barner Lekdorf, Kajsa Kvist, John B. Buse, on behalf of the DEVOTE Study Group

**Affiliations:** 1 Lunenfeld-Tanenbaum Research Institute, Mount Sinai Hospital, 60 Murray St, Box 17, University of Toronto, Toronto, ON M5T 3L9 Canada; 2 Research Medical Center, Kansas City, MO USA; 3 Imperial Clinical Trials Unit, Imperial College London, London, UK; 4 University of Washington, Seattle, WA USA; 5 Medical University of Graz, Graz, Austria; 6 Florida Hospital Translational Research Institute for Metabolism and Diabetes, Orlando, FL USA; 7 Sanford Burnham Prebys Medical Discovery Institute, Orlando, FL USA; 8 Novo Nordisk A/S, Søborg, Denmark; 9 University of North Carolina School of Medicine, Chapel Hill, NC USA

**Keywords:** Hypoglycaemia, Insulin therapy, Macrovascular disease

## Abstract

**Aims/hypothesis:**

The Trial Comparing Cardiovascular Safety of Insulin Degludec vs Insulin Glargine in Patients with Type 2 Diabetes at High Risk of Cardiovascular Events (DEVOTE) was a double-blind, randomised, event-driven, treat-to-target prospective trial comparing the cardiovascular safety of insulin degludec with that of insulin glargine U100 (100 units/ml) in patients with type 2 diabetes at high risk of cardiovascular events. This paper reports a secondary analysis investigating associations of day-to-day fasting glycaemic variability (pre-breakfast self-measured blood glucose [SMBG]) with severe hypoglycaemia and cardiovascular outcomes.

**Methods:**

In DEVOTE, patients with type 2 diabetes were randomised to receive insulin degludec or insulin glargine U100 once daily. The primary outcome was the first occurrence of an adjudicated major adverse cardiovascular event (MACE). Adjudicated severe hypoglycaemia was the pre-specified secondary outcome. In this article, day-to-day fasting glycaemic variability was based on the standard deviation of the pre-breakfast SMBG measurements. The variability measure was calculated as follows. Each month, only the three pre-breakfast SMBG measurements recorded before contact with the site were used to determine a day-to-day fasting glycaemic variability measure for each patient. For each patient, the variance of the three log-transformed pre-breakfast SMBG measurements each month was determined. The standard deviation was determined as the square root of the mean of these monthly variances and was defined as day-to-day fasting glycaemic variability. The associations between day-to-day fasting glycaemic variability and severe hypoglycaemia, MACE and all-cause mortality were analysed for the pooled trial population with Cox proportional hazards models. Several sensitivity analyses were conducted, including adjustments for baseline characteristics and most recent HbA_1c_.

**Results:**

Day-to-day fasting glycaemic variability was significantly associated with severe hypoglycaemia (HR 4.11, 95% CI 3.15, 5.35), MACE (HR 1.36, 95% CI 1.12, 1.65) and all-cause mortality (HR 1.58, 95% CI 1.23, 2.03) before adjustments. The increased risks of severe hypoglycaemia, MACE and all-cause mortality translate into 2.7-, 1.2- and 1.4-fold risk, respectively, when a patient’s day-to-day fasting glycaemic variability measure is doubled. The significant relationships of day-to-day fasting glycaemic variability with severe hypoglycaemia and all-cause mortality were maintained after adjustments. However, the significant association with MACE was not maintained following adjustment for baseline characteristics with either baseline HbA_1c_ (HR 1.19, 95% CI 0.96, 1.47) or the most recent HbA_1c_ measurement throughout the trial (HR 1.21, 95% CI 0.98, 1.49).

**Conclusions/interpretation:**

Higher day-to-day fasting glycaemic variability is associated with increased risks of severe hypoglycaemia and all-cause mortality.

**Trial registration:**

ClinicalTrials.gov NCT01959529

## Introduction

The main goal of glucose-lowering therapy in type 2 diabetes is to reduce the incidence and progression of both microvascular and macrovascular complications [1]. However, while insulin is traditionally considered to be the most effective glucose-lowering therapy, and often becomes necessary as type 2 diabetes progresses, it is associated with an increased risk of hypoglycaemia [2, 3]. Hypoglycaemia has far-reaching consequences, and can significantly reduce a patient’s (and their family’s) quality of life. Concern about increasing a patient’s risk of hypoglycaemia can also have a negative impact on the treatment decisions made by physicians [2–5]. Moreover, there is evidence that hypoglycaemia may be associated with adverse cardiovascular outcomes and mortality, thereby further impacting the goals of therapy [6–10].

Although the risk of hypoglycaemia is influenced by multiple factors, unstable glycaemic control – including high day-to-day glucose variability (i.e. the variation in glucose level at a given time point(s) over a series of days) – is associated with a higher incidence of hypoglycaemic events [11–13]. Glycaemic variability is also associated with the development of other diabetes-related complications based on evidence from several previous studies where important outcomes such as the risks of severe hypoglycaemia and cardiovascular events were associated not only with average blood glucose levels, but also with glycaemic variability [11–15]. However, these observations have not been consistent across studies and require further investigation [16–19].

Insulin degludec is an ultra-long-acting (pharmacokinetic half-life of 25 h) basal insulin designed to achieve a highly stable pharmacodynamic profile, with lower within-day and day-to-day variability in its glucose-lowering action compared with insulin glargine U100 (100 units/ml) and glargine U300 (300 units/ml). These properties have been demonstrated across different patient populations in several experimental laboratory studies [20, 21]. In the clinical setting, insulin degludec has consistently demonstrated lower rates of overall confirmed, nocturnal confirmed and severe hypoglycaemia compared with other basal insulins [22–24].

The cardiovascular safety of insulin degludec compared with insulin glargine U100 has recently been studied in the Double-blinded Trial Comparing Cardiovascular Safety of Insulin Degludec vs Insulin Glargine in Patients with Type 2 Diabetes at High Risk of Cardiovascular Events (DEVOTE) [24, 25]. The pre-specified primary analysis demonstrated that insulin degludec was non-inferior to insulin glargine in terms of cardiovascular events (HR 0.91, 95% CI 0.78, 1.06), and superior with regard to hypoglycaemia risk, with lower rates of both severe and nocturnal severe hypoglycaemia (by 40% and 53%, respectively; both *p* < 0.001), achieved at similar levels of glycaemic control as assessed by HbA_1c_ [24]. Because of the size and design of the trial, DEVOTE provides a valuable opportunity to investigate the relationships of day-to-day fasting glycaemic variability with important outcomes such as severe hypoglycaemia, major adverse cardiovascular events (MACEs) and all-cause mortality.

## Methods

The detailed methods of the trial, the trial protocol, the statistical analysis plan and the list of members of the trial teams and committees have been published previously [24, 25]. DEVOTE is registered at ClinicalTrials.gov (number NCT01959529). The trial was conducted in accordance with the Declaration of Helsinki and ICH Good Clinical Practice Guideline [26, 27]. The protocol was approved by independent ethics committees or institutional review boards for each centre; written informed consent was obtained from each patient before any trial-related activities.

In brief, DEVOTE was a multicentre, prospective, treat-to-target, randomised, double-blind, active-comparator cardiovascular outcomes trial designed to continue until at least 633 MACEs, confirmed by a central, blinded, independent Event Adjudication Committee (EAC), had accrued [24, 25]. All participants had type 2 diabetes treated with at least one oral or injectable glucose-lowering agent with HbA_1c_ ≥ 7.0% (53 mmol/mol), or treated with ≥ 20 units/day of basal insulin. Patients were eligible for the trial if they either had at least one co-existing cardiovascular or renal condition and were aged ≥ 50 years, or had at least one of a list of pre-specified cardiovascular risk factors and were aged ≥ 60 years.

Patients with type 2 diabetes at high risk of cardiovascular events were randomised 1:1 to receive either insulin degludec (Novo Nordisk, Bagsværd, Denmark) or insulin glargine (Sanofi, Paris, France), both in identical 100 U/ml, 10 ml vials, administered once daily between dinner and bedtime, in addition to standard care. All patients were allowed to continue their pre-trial glucose-lowering therapy with the exception of basal and premix insulins, which were discontinued.

Patients were to titrate their basal insulin weekly, based on the lowest of three pre-breakfast self-measured blood glucose (SMBG) values measured 2 days before and on the day of titration, with the aim of achieving a target of 4.0–5.0 mmol/l. To safeguard vulnerable patients, an alternative target of 5.0–7.0 mmol/l was permitted at the discretion of the investigator. Bolus insulin (insulin aspart), provided by Novo Nordisk for patients either continuing or initiating bolus treatment during the trial, was to be titrated weekly based on the lowest of three preprandial or bedtime SMBG values measured on the 3 days before titration, with the aim being to achieve a target of 4.0–7.0 mmol/l. Higher targets were allowed at the discretion of the investigator.

The following events were adjudicated by the EAC in a blinded manner: severe hypoglycaemia, acute coronary syndrome (defined as myocardial infarction or unstable angina pectoris requiring hospitalisation), stroke and fatal events. The primary composite endpoint was the time from randomisation to the first occurrence of death from cardiovascular causes, non-fatal myocardial infarction or non-fatal stroke. Severe hypoglycaemia was a pre-specified, multiplicity-adjusted secondary outcome, as defined by the ADA as an episode requiring the assistance of another person to actively administer carbohydrate or glucagon, or to take other corrective actions. Plasma glucose levels may not be available during an event, but neurological recovery is considered sufficient evidence that the event was induced by a low plasma glucose level [28].

In this secondary analysis, the standard deviation of the pre-breakfast SMBG measurements, defined as the day-to-day fasting glycaemic variability measure, was calculated as follows. Each month, only the three pre-breakfast SMBG measurements recorded before contact with the site were used to determine a day-to-day fasting glycaemic variability measure for each patient. For each patient, the variance of the three log-transformed (natural logarithm) pre-breakfast SMBG measurements each month was determined. The standard deviation was determined as the square root of the mean of these monthly variances and was defined as day-to-day fasting glycaemic variability. For descriptive purposes, this measure of glycaemic variability is expressed as the geometric coefficient of variation, a measure of dispersion relative to the geometric mean, corresponding to 1 standard deviation dispersion around the geometric mean [29]. It is computed by exponentiating the standard deviation of the log-transformed pre-breakfast SMBG measurements and subtracting 1. Day-to-day fasting glycaemic variability was calculated on a logarithmic scale in order to reduce the interdependency between mean glycaemic control and measures of day-to-day fasting glycaemic variability. An investigation with interaction terms indicated that the association between day-to-day fasting glycaemic variability and outcomes was the same for both treatment arms (insulin degludec and insulin glargine), and so the association is reported for the pooled population. All analyses were conducted using SAS, version 9.4 (https://www.sas.com/en_ca/software/sas9.html). A *p* value of < 0.05 was considered statistically significant.

**Fig. 1:**
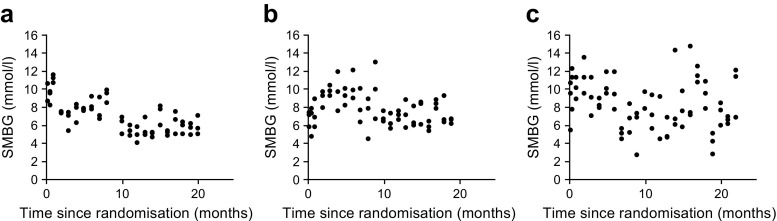
Representative SMBG profiles from three separate DEVOTE participants illustrating the low (**a**), medium (**b**) and high (**c**) variability groups. Day-to-day fasting glycaemic variability was based on the standard deviation of the pre-breakfast SMBG measurements

For the purposes of summarising the baseline characteristics by low and high day-to-day fasting glycaemic variability, the patient population was divided into tertiles or thirds defined by low, medium and high variability. Grouping patients into thirds to investigate variability has been used in previous studies [30–32]. Example SMBG profiles for three separate DEVOTE patients can be seen in Fig. 1. These three patients represent each of the three variability groups. Each variability group represents relative values based on the range of variability within a given trial, and thus should be interpreted only within a given trial.

The associations of day-to-day fasting glycaemic variability with time to first severe hypoglycaemic event, time to first MACE and time to all-cause mortality were investigated with Cox proportional hazards models. The models were adjusted for treatment and baseline laboratory-measured fasting plasma glucose (FPG). Several sensitivity analyses were conducted. The first analysis adjusted for the most recent HbA_1c_ measurement as a time-dependent covariate. The second analysis, as was reported in Marso et al [24], adjusted for investigational product, baseline HbA_1c_ above or below 8% (64 mmol/mol), sex, region, age at baseline, smoking status at baseline, diabetes duration at baseline, cardiovascular risk group inclusion criteria, insulin-naive at baseline and renal function (eGFR) at baseline. This analysis was then repeated twice, either adjusting for baseline HbA_1c_ on a continuous scale or using the most recent HbA_1c_ measurement as a time-dependent covariate.

## Results

A total of 7637 patients were randomised to either insulin degludec (*n* = 3818) or insulin glargine (*n* = 3819). Of these, 93% completed the final follow-up visit. Vital status was known for 99.9% of patients. The median observation time was 1.99 years. Of the 7637 randomised participants, a variability measure could not be established for 51 of these participants.

### Baseline characteristics and outcomes by variability group

Baseline characteristics differed across the three glycaemic variability groups. In particular, the high variability group had a longer duration of diabetes (18.8 years vs 14.1 [low variability group] and 16.3 [medium variability group] years), higher HbA_1c_ levels (8.8% [72.2 mmol/mol] vs 8.1% [65.4 mmol/mol; low variability group] and 8.4% [68.2 mmol/mol; medium variability group]), higher FPG levels (9.9 mmol/l vs 9.2 [low variability group] and 9.5 [medium variability group]), and lower eGFRs (64.7 ml^−1^ min^−1^ (1.73 m)^−2^ vs 70.5 [low variability group] and 68.7 [medium variability group]) (Table 1). The three variability groups were similar in terms of lipid levels, body weight, blood pressure, cardiovascular risk group inclusion criteria and smoking status. The mean, standard deviation, and minimum and maximum levels of variability for each variability group are summarised in Table 2, along with the HbA_1c_ levels at baseline and month 24. Overall, the average proportionate change in HbA_1c_ levels from baseline to month 24 was similar across the three variability groups. A summary of the numbers and rates of events by variability group for severe hypoglycaemia, MACE (including the individual components of the MACE composite) and all-cause mortality are included in Table 3. There was no between-treatment difference in terms of the association between day-to-day fasting glycaemic variability and the risk of severe hypoglycaemia, MACE or all-cause mortality. On this basis, the association between day-to-day fasting glycaemic variability and outcomes is reported for the pooled population.

**Table 1:** Baseline characteristics by variability group

Characteristic	Low variability *n* = 2528	Medium variability *n* = 2530	High variability *n* = 2528
Age (years)	64.7 ± 7.4^a^	65.0 ± 7.3^b^	65.3 ± 7.4
Patients aged ≥ 75 years	261 (10.3)	262 (10.4)	284 (11.2)
Men	1617 (64.0)	1621 (64.1)	1515 (59.9)
Ethnicity			
Hispanic or Latino	310 (12.3)	356 (14.1)	465 (18.4)
Race			
White	1873 (74.1)	1948 (77.0)	1919 (75.9)
Black or African-American	199 (7.9)	270 (10.7)	355 (14.0)
Asian	389 (15.4)	229 (9.1)	155 (6.1)
Other	67 (2.7)	83 (3.3)	99 (3.9)
Region			
North America	1506 (59.6)	1760 (69.6)	1973 (78.0)
Europe	456 (18.0)	278 (11.0)	131 (5.2)
South America	143 (5.7)	194 (7.7)	247 (9.8)
India	204 (8.1)	100 (4.0)	51 (2.0)
Asia excluding India	136 (5.4)	95 (3.8)	60 (2.4)
Africa	83 (3.3)	103 (4.1)	66 (2.6)
Diabetes duration (years)	14.1 ± 8.1	16.3 ± 8.6	18.8 ± 9.3
Smoking status			
Current	251 (9.9)	276 (10.9)	321 (12.7)
Previous	1096 (43.4)	1147 (45.3)	1093 (43.2)
Never	1181 (46.7)	1107 (43.8)	1114 (44.1)
Trial eligibility stratum			
Age ≥ 50 years and established cardiovascular or chronic kidney disease^c^	2147 (84.9)	2148 (84.9)	2172 (85.9)
Age ≥ 60 years and risk factors for cardiovascular disease^d^	371 (14.7)	377 (14.9)	351 (13.9)
Body weight (kg)	95.9 ± 22.8	97.2 ± 23.3	95.3 ± 22.5
BMI (kg/m^2^)	33.5 ± 6.7	33.8 ± 6.9	33.4 ± 6.9
Blood pressure			
Systolic (mmHg)	134.7 ± 17.1	136.3 ± 18.1	135.6 ± 18.8
Diastolic (mmHg)	76.9 ± 10.0	76.5 ± 10.3	75.1 ± 10.6
Pulse (beats/min)	73.2 ± 11.2	73.1 ± 11.4	73.0 ± 11.4
HbA_1c_ (%)	8.1 ± 1.6	8.4 ± 1.6	8.8 ± 1.7
HbA_1c_ (mmol/mol)	65.4 ± 17.3	68.2 ± 17.5	72.2 ± 18.6
FPG (mmol/l)	9.2 ± 3.5	9.5 ± 3.7	9.9 ± 4.4
eGFR (ml^−1^ min^−1^ [1.73 m]^−2^) based on CKD-EPI	70.5 ± 21.1	68.7 ± 21.3	64.7 ± 21.8
Total cholesterol (mmol/l)	4.3 ± 1.2	4.2 ± 1.2	4.3 ± 1.2
LDL-cholesterol (mmol/l)	2.2 ± 0.9	2.2 ± 0.9	2.2 ± 1.0
HDL-cholesterol (mmol/l)	1.1 ± 0.3	1.1 ± 0.3	1.2 ± 0.4
Triacylglycerols^e^ (mmol/l)	2.1 ± 2.0	2.1 ± 1.6	2.0 ± 1.8

Full analysis set (all randomised patients); data listed are number (proportion [%]) or mean ± SD. Percentage refers to the proportion of patients on degludec or glargine treatment

^a^Including two patients aged < 50 years

^b^Including one patient aged < 50 years

^c^Patients with missing age information or aged < 50 years, but who fulfilled at least one of the inclusion criteria for established cardiovascular/chronic kidney disease were included

^d^Patients with missing age information and who only fulfilled the inclusion criteria for cardiovascular disease risk factors were not included

^e^Triacylglycerols is equivalent to triglycerides

CKD-EPI, chronic kidney disease epidemiology collaboration formula

**Table 2:** Variability and HbA_1c_ levels by variability group

Variability/HbA_1c_	Low variability *n* = 2528	Medium variability *n* = 2530	High variability *n* = 2528
Variability^a^			
Mean ± SD	14 ± 3%	23 ± 2%	36 ± 9%
Min, Max	1%, 19%	19%, 27%	27%, 138%
Mean number of monthly variances^b^	22.2 ± 5.7	22.6 ± 5.3	22.2 ± 5.8
Mean number of blood glucose measurements^b^			
Week 1	2.9 ± 0.4	2.9 ± 0.4	2.9 ± 0.5
Month 12	2.9 ± 0.4	2.9 ± 0.3	2.9 ± 0.5
Month 24	2.8 ± 0.6	2.9 ± 0.6	2.8 ± 0.7
HbA_1c_ at baseline^b^ (%)	8.1 ± 1.6	8.4 ± 1.6	8.8 ± 1.7
HbA_1c_ at baseline^b^ (mmol/mol)	65.4 ± 17.3	68.2 ± 17.5	72.2 ± 18.6
HbA_1c_ at 24 months^b^ (%)	7.2 ± 1.2	7.4 ± 1.2	7.8 ± 1.2
HbA_1c_ at 24 months^b^ (mmol/mol)	55.5 ± 12.8	57.8 ± 12.7	61.7 ± 13.1
Change in HbA_1c_ from baseline to 24 months (%)	−0.8 ± 1.4	−0.9 ± 1.6	−0.8 ± 1.6
Change in HbA_1c_ from baseline to 24 months (mmol/mol)	−8.6 ± 15.8	−10.0 ± 17.2	−9.3 ± 17.5

^a^Variability is described as the geometric coefficient of variation, corresponding to 1 SD dispersion around the geometric mean. It is computed by exponentiating the SD of the log SMBG and subtracting 1

^b^Data are mean ± SD

**Table 3:** Outcomes by variability group

Outcome	Low variability *n* = 2528		Medium variability *n* = 2530		High variability *n* = 2528	
	Events	Rate	Events	Rate	Events	Rate
Severe hypoglycaemia	83	1.69	116	2.38	237	5.00
MACE	187	3.84	219	4.49	267	5.48
Cardiovascular death	75	1.50	83	1.65	116	2.30
Non-fatal MI	90	1.83	104	2.11	117	2.37
Non-fatal stroke	37	0.75	50	1.00	61	1.23
All-cause mortality	115	2.30	131	2.61	171	3.40

MI, myocardial infarction; rate, events per 100 patient-years of observation

### Associations between day-to-day fasting glycaemic variability and outcomes

**Fig. 2:**
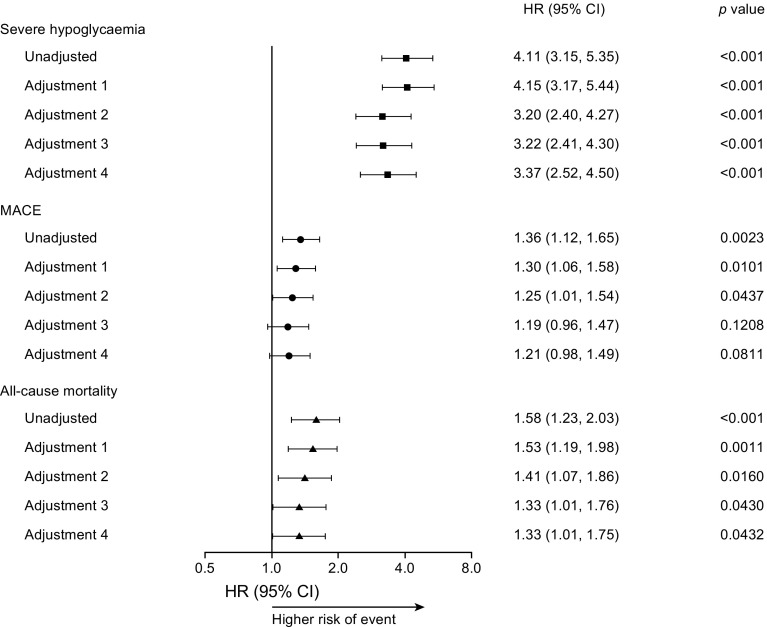
Day-to-day fasting glycaemic variability and its associations with severe hypoglycaemia, MACE and all-cause mortality. Day-to-day fasting glycaemic variability was based on the standard deviation of the pre-breakfast SMBG measurements. Adjustment 1: adjusted for the most recent HbA_1c_ measurement on a continuous scale. Adjustment 2: adjusted for baseline HbA_1c_ above or below 8% (64 mmol/mol) and baseline characteristics (investigational product, sex, region, age at baseline, smoking status at baseline, diabetes duration at baseline, cardiovascular risk group inclusion criteria, insulin-naive at baseline and renal function [eGFR] at baseline). Adjustment 3: adjusted for baseline HbA_1c_ on a continuous scale and baseline characteristics as for adjustment 2. Adjustment 4: adjusted for most recent HbA_1c_ measurement on a continuous scale and baseline characteristics as for adjustment 2

**Fig. 3:**
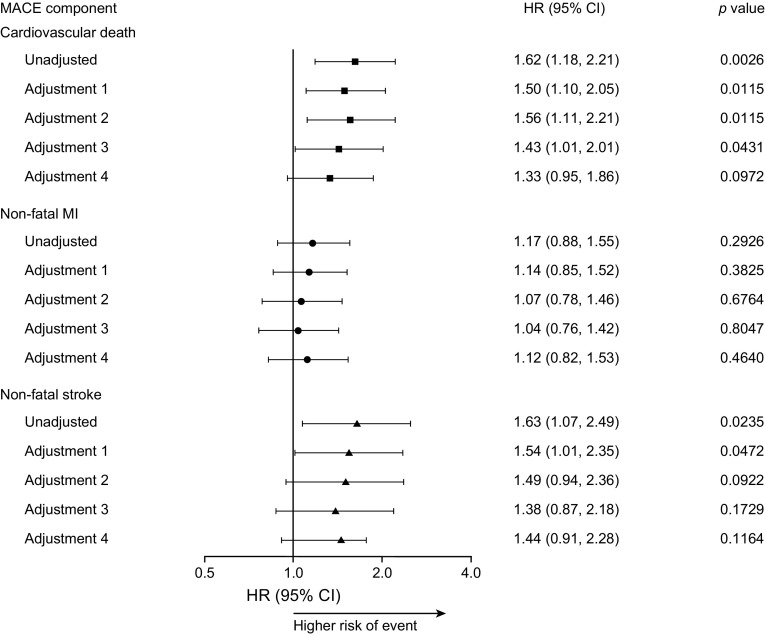
Day-to-day fasting glycaemic variability and its association with cardiovascular death, non-fatal myocardial infarction and non-fatal stroke. Day-to-day fasting glycaemic variability was based on the standard deviation of the pre-breakfast SMBG measurements. For adjustments, see Fig. 2 legend. MI, myocardial infarction

Higher day-to-day fasting glycaemic variability was significantly associated with higher risks of severe hypoglycaemia (HR 4.11, *p* < 0.001), MACE (HR 1.36, *p* = 0.0023) and all-cause mortality (HR 1.58, *p* < 0.001) before adjustments (Fig. 2). The increased risks of severe hypoglycaemia, MACE and all-cause mortality translate into 2.7-, 1.2- and 1.4-fold risk, respectively, when a patient’s day-to-day fasting glycaemic variability measure is doubled. The significant relationship between day-to-day fasting glycaemic variability and severe hypoglycaemia and all-cause mortality was maintained after adjustments for the most recent HbA_1c_ measurement throughout the trial (HR 4.15, *p* < 0.0001; HR 1.53, *p* = 0.0011) or baseline characteristics (investigational product, sex, region, age at baseline, smoking status at baseline, diabetes duration at baseline, cardiovascular risk group inclusion criteria, insulin-naive at baseline and renal function [eGFR] at baseline) with either baseline HbA_1c_ above or below 8% (64 mmol/mol; HR 3.20, *p* < 0.001; HR 1.41, *p* = 0.0160), baseline HbA_1c_ (HR 3.22, *p* < 0.001; HR 1.33, *p* = 0.0430) or the most recent HbA_1c_ measurement throughout the trial (HR 3.37, *p* < 0.001; HR 1.33, *p* = 0.0432) (Fig. 2). The significant association with MACE before adjustment was maintained after adjustments for the most recent HbA_1c_ measurement throughout the trial (HR 1.30, *p* = 0.0101) or baseline characteristics with baseline HbA_1c_ above or below 8% (64 mmol/mol; HR 1.25, *p* = 0.0437) (Fig. 2). However, the significant association was not maintained following adjustment for baseline characteristics with either baseline HbA_1c_ (HR 1.19, *p* = 0.1208) or the most recent HbA_1c_ measurement throughout the trial (HR 1.21, *p* = 0.0811; Fig. 2). The associations of day-to-day fasting glycaemic variability with the individual components of the MACE composite are shown in Fig. 3.

## Discussion

The results of the analyses presented here indicate associations of day-to-day fasting with glycaemic variability, severe hypoglycaemia and all-cause mortality.

A well-accepted consequence of high glycaemic variability is that individuals with diabetes are more likely to experience hypoglycaemia, and in particular severe hypoglycaemia, as demonstrated by several studies [11–13, 33–35]. A prospective observational study using continuous glucose monitoring reported a significant association between higher standard deviation of mean glucose and a higher incidence of asymptomatic hypoglycaemia [34]. The Diabetes Outcomes in Veterans Study also found that mean blood glucose and the standard deviation of mean glucose were stronger determinants of hypoglycaemia than HbA_1c_ [35]. Furthermore, a pooled analysis demonstrated that the day-to-day glucose coefficient of variation was significantly correlated with the rate of hypoglycaemia [36]. It is important to note, however, that there are several metrics for assessing glycaemic variability. The above studies focused on mean glycaemic variability, while in our analysis we focused on the fasting SMBG measurements as a measure of day-to-day fasting glycaemic variability. It is currently unknown whether fasting blood glucose variability confers additional risk for adverse events beyond those associated with chronic hyperglycaemia. In this context, our study clearly demonstrates that higher day-to-day fasting glycaemic variability is associated with a higher risk of both severe hypoglycaemia and all-cause mortality. This result was consistent even following adjustments for various baseline characteristics, including baseline HbA_1c_ and the most recent HbA_1c_ measurement throughout the trial. A similar result was reported by an analysis of the Predictable Results and Experience in Diabetes through Intensification and Control to Target: An International Variability Evaluation (PREDICTIVE) study that used FPG values as a measure of variability, whereby fasting glycaemic variability was significantly associated with nocturnal hypoglycaemia following adjustments for change in HbA_1c_ measurements [37].

Clamp studies have shown that fluctuations in blood glucose induce higher levels of oxidative stress and endothelial dysfunction, factors that are implicated in the development of cardiovascular disease in individuals with type 2 diabetes, compared with stable, constant high blood glucose concentrations [38–40]. However, it is possible that glycaemic variability may indirectly increase the risk of cardiovascular events due to an increase in severe hypoglycaemia for which evidence of an association with cardiovascular events is accumulating [41, 42].

Irrespective of this, high fasting glycaemic variability and hypoglycaemia are concerns for all patients treated with blood glucose-lowering therapies. In addition, our results also raise the possibility that there are some patients who are more susceptible to severe hypoglycaemia because they are unable or unwilling, for a variety of reasons, to appropriately modify their insulin dose to reduce their fasting glycaemic variability. On this basis, the findings from the current study provide further support for the concept that patients requiring insulin might benefit from treatment with a basal insulin that has low day-to-day variability and hence provides consistent fasting blood glucose levels.

A limitation of this study was that glycaemic variability was related solely to fasting SMBG values, albeit these were measured frequently during the trial. In addition, for each participant, one measure of variability was determined based on SMBG measurements recorded during the whole trial and included in the analysis as a baseline variable. A possible improvement could have been to update each patient’s variability measure throughout the trial because variability may have changed as glycaemic stability was achieved.

The present analyses have a number of strengths, including the large sample size, the double-blind active-control design, the high level of cardiovascular risk of the patient population, and the independent adjudication of cardiovascular and severe hypoglycaemic events. The prospective design and multicentre, international nature of this trial and the high levels of patient follow-up further contributed to the robustness of the analyses. In addition, while other measures of glucose variability could have been used, the method we have used in our analyses accounts for variation in fasting SMBG values resulting from the changing dose of basal insulin during the titration phase. By first determining the monthly variances for each patient and then taking the mean, this allows the day-to-day variability to be separated from the overall mean glycaemic control, especially as the latter may be affected by the treat-to-target regimen. Further contributing to our ability to separate effects of mean glycaemic control and day-to-day glycaemic variability was the use of relative comparisons effected by analysing the data on a logarithmic scale. An additional strength of this method was the high number of SMBG measurements used to determine each patient’s day-to-day variability, thereby strengthening our assessment of variability. Furthermore, the inclusion of several sensitivity analyses provides additional information about the relationship between day-to-day fasting glycaemic variability and outcomes. These analyses demonstrated that the significant association between day-to-day fasting glycaemic variability and MACE was lost following adjustment for baseline characteristics, some of which are known to be associated with increased cardiovascular risk, and either baseline HbA_1c_ or the most recent HbA_1c_ measurement throughout the trial. Of note, however, significance was maintained when only adjusted for the most recent HbA_1c_ measurement throughout the trial. It is likely that the significant association is lost in part due to multicollinearity of baseline factors related to MACE. For example, diabetes duration and region are included in the multivariable adjustments and are non-significant predictors. However, if either is deleted from the multivariable adjustments, the association between day-to-day fasting glycaemic variability and MACE remains significant.

In conclusion, evidence from DEVOTE supports associations between higher day-to-day fasting glycaemic variability and increased risks of severe hypoglycaemia and all-cause mortality.

## Abbreviations

DEVOTE: Trial Comparing Cardiovascular Safety of Insulin Degludec vs Insulin Glargine in Patients with Type 2 Diabetes at High Risk of Cardiovascular Events
EAC: Event Adjudication Committee
FPG: Fasting plasma glucose
MACE: Major adverse cardiovascular event
SMBG: Self-measured blood glucose

## References

[1] Dailey G (2011). Overall mortality in diabetes mellitus: where do we stand today?. Diabetes Technol Ther.

[2] Fidler C, Elmelund Christensen T, Gillard S (2011). Hypoglycemia: an overview of fear of hypoglycemia, quality-of-life, and impact on costs. J Med Econ.

[3] Frier BM (2008). How hypoglycaemia can affect the life of a person with diabetes. Diabetes Metab Res Rev.

[4] Brod M, Galstyan G, Unnikrishnan AG (2016). Self-treated hypoglycemia in type 2 diabetes mellitus: results from the second wave of an international cross-sectional survey. Diabetes Ther.

[5] Peyrot M, Barnett AH, Meneghini LF, Schumm-Draeger PM (2012). Insulin adherence behaviours and barriers in the multinational Global Attitudes of Patients and Physicians in Insulin Therapy study. Diabet Med.

[6] Johnston SS, Conner C, Aagren M, Smith DM, Bouchard J, Brett J (2011). Evidence linking hypoglycemic events to an increased risk of acute cardiovascular events in patients with type 2 diabetes. Diabetes Care.

[7] Skyler JS, Bergenstal R, Bonow RO (2009). Intensive glycemic control and the prevention of cardiovascular events: implications of the ACCORD, ADVANCE, and VA Diabetes Trials: a position statement of the American Diabetes Association and a Scientific Statement of the American College of Cardiology Foundation and the American Heart Association. Circulation.

[8] Cha SA, Yun JS, Lim TS (2016). Severe hypoglycemia and cardiovascular or all-cause mortality in patients with type 2 diabetes. Diabetes Metab J.

[9] Zoungas S, Patel A, Chalmers J, et al. ADVANCE Collaborative Group (2010) Severe hypoglycemia and risks of vascular events and death. N Engl J Med 363:1410–1418 10.1056/NEJMoa1003795 20925543

[10] Khunti K, Davies M, Majeed A, Thorsted BL, Wolden ML, Paul SK (2015). Hypoglycemia and risk of cardiovascular disease and all-cause mortality in insulin-treated people with type 1 and type 2 diabetes: a cohort study. Diabetes Care.

[11] Hirakawa Y, Arima H, Zoungas S (2014). Impact of visit-to-visit glycemic variability on the risks of macrovascular and microvascular events and all-cause mortality in type 2 diabetes: the ADVANCE trial. Diabetes Care.

[12] Bailey TS, Bhargava A, Hans de Vries J (2017). Day-to-day variability of fasting self-measured plasma glucose (SMPG) correlates with risk of hypoglycemia in adults with type 1 (T1D) and type 2 diabetes (T2D). Diabetes.

[13] Miller ME, Bonds DE, Gerstein HC (2010). The effects of baseline characteristics, glycaemia treatment approach, and glycated haemoglobin concentration on the risk of severe hypoglycaemia: post hoc epidemiological analysis of the ACCORD study. BMJ.

[14] Stratton IM, Adler AI, Neil HA (2000). Association of glycaemia with macrovascular and microvascular complications of type 2 diabetes (UKPDS 35): prospective observational study. BMJ.

[15] Heller SR, Bergenstal RM, White WB (2017). Relationship of glycated haemoglobin and reported hypoglycaemia to cardiovascular outcomes in patients with type 2 diabetes and recent acute coronary syndrome events: the EXAMINE trial. Diabetes Obes Metab.

[16] Ceriello A, Kilpatrick ES (2013). Glycemic variability: both sides of the story. Diabetes Care.

[17] Frontoni S, Di Bartolo P, Avogaro A, Bosi E, Paolisso G, Ceriello A (2013). Glucose variability: an emerging target for the treatment of diabetes mellitus. Diabetes Res Clin Pract.

[18] Lin CC, Li CI, Yang SY (2012). Variation of fasting plasma glucose: a predictor of mortality in patients with type 2 diabetes. Am J Med.

[19] Smith-Palmer J, Brändle M, Trevisan R, Orsini Federici M, Liabat S, Valentine W (2014). Assessment of the association between glycemic variability and diabetes-related complications in type 1 and type 2 diabetes. Diabetes Res Clin Pract.

[20] Haahr H, Heise T (2014). A review of the pharmacological properties of insulin degludec and their clinical relevance. Clin Pharmacokinet.

[21] Heise T, Nørskov M, Nosek L, Kaplan K, Famulla S, Haahr HL (2017). Insulin degludec: lower day-to-day and within-day variability in pharmacodynamic response compared to insulin glargine U300 in type 1 diabetes. Diabetes Obes Metab.

[22] Ratner RE, Gough SC, Mathieu C (2013). Hypoglycaemia risk with insulin degludec compared with insulin glargine in type 2 and type 1 diabetes: a pre-planned meta-analysis of phase 3 trials. Diabetes Obes Metab.

[23] Wysham C, Bhargava A, Chaykin L (2017). Effect of insulin degludec vs insulin glargine U100 on hypoglycemia in patients with type 2 diabetes: the SWITCH 2 randomized clinical trial. JAMA.

[24] Marso SP, McGuire DK, Zinman B et al (2017) Efficacy and safety of degludec versus glargine in type 2 diabetes. N Engl J Med 377:723–732 10.1056/NEJMoa1615692 PMC5731244 28605603

[25] Marso SP, McGuire DK, Zinman B (2016). Design of DEVOTE (trial comparing cardiovascular safety of insulin degludec vs insulin glargine in patients with type 2 diabetes at high risk of cardiovascular events)—DEVOTE 1. Am Heart J.

[26] World Medical Association. Declaration of Helsinki (2013). Ethical principles for medical research involving human subjects. JAMA.

[27] ICH harmonised tripartite guideline: guideline for good clinical practice (2001) J Postgrad Med 47:199–203 11832625

[28] Seaquist ER, Anderson J, Childs B (2013). Hypoglycemia and diabetes: a report of a workgroup of the American Diabetes Association and the Endocrine Society. Diabetes Care.

[29] Kirkwood TBL (1979). Geometric means and measures of dispersion. Biometrics.

[30] Crenier L, Abou-Elias C, Corvilain B (2013). Glucose variability assessed by low blood glucose index is predictive of hypoglycemic events in patients with type 1 diabetes switched to pump therapy. Diabetes Care.

[31] Takao T, Matsuyama Y, Yanagisawa H, Kikuchi M, Kawazu S (2014). Association between HbA1c variability and mortality in patients with type 2 diabetes. J Diabetes Complications.

[32] Akrivos J, Ravona-Springer R, Schmeidler J (2015). Glycemic control, inflammation, and cognitive function in older patients with type 2 diabetes. Int J Geriatr Psychiatry.

[33] Cox DJ, Kovatchev BP, Julian DM (1994). Frequency of severe hypoglycemia in insulin-dependent diabetes mellitus can be predicted from self-monitoring blood glucose data. J Clin Endocrinol Metab.

[34] Monnier L, Wojtusciszyn A, Colette C, Owens D (2011). The contribution of glucose variability to asymptomatic hypoglycemia in persons with type 2 diabetes. Diabetes Technol Ther.

[35] Murata GH, Hoffman RM, Shah JH, Wendel CS, Duckworth WC (2004). A probabilistic model for predicting hypoglycemia in type 2 diabetes mellitus: the Diabetes Outcomes in Veterans Study (DOVES). Arch Intern Med.

[36] Qu Y, Jacober SJ, Zhang Q, Wolka LL, DeVries JH (2012). Rate of hypoglycemia in insulin-treated patients with type 2 diabetes can be predicted from glycemic variability data. Diabetes Technol Ther.

[37] Niskanen L, Virkamäki A, Hansen JB, Saukkonen T (2009). Fasting plasma glucose variability as a marker of nocturnal hypoglycemia in diabetes: evidence from the PREDICTIVE study. Diabetes Res Clin Pract.

[38] Ceriello A, Esposito K, Piconi L (2008). Oscillating glucose is more deleterious to endothelial function and oxidative stress than mean glucose in normal and type 2 diabetic patients. Diabetes.

[39] Esper RJ, Nordaby RA, Vilarino JO, Paragano A, Cacharrón JL, Machado RA (2006). Endothelial dysfunction: a comprehensive appraisal. Cardiovasc Diabetol.

[40] Ceriello A, Taboga C, Tonutti L (2002). Evidence for an independent and cumulative effect of postprandial hypertriglyceridemia and hyperglycemia on endothelial dysfunction and oxidative stress generation: effects of short- and long-term simvastatin treatment. Circulation.

[41] Gerstein HC, Miller ME, Byington RP (2008). Effects of intensive glucose lowering in type 2 diabetes. N Engl J Med.

[42] Duckworth W, Abraira C, Moritz T (2009). Glucose control and vascular complications in veterans with type 2 diabetes. N Engl J Med.

